# Longitudinal brain connectivity changes associated with successful smoking cessation

**DOI:** 10.3389/fpsyg.2025.1734803

**Published:** 2025-12-17

**Authors:** Jooyeon Jamie Im, Hyeonjin Kim, Jeung-Hyun Lee, Hyung Jun Park, Hee-Kyung Joh, Woo-Young Ahn

**Affiliations:** 1 Department of Psychology, Seoul National University, Seoul, Republic of Korea; 2 Center for Health Promotion, Samsung Medical Center, Seoul, Republic of Korea; 3 Department of Clinical Medical Sciences, College of Medicine, Seoul National University, Seoul, Republic of Korea; 4 Department of Family Medicine, Seoul National University Health Service Center, Seoul, Republic of Korea; 5 Department of Medicine, Seoul National University College of Medicine, Seoul, Republic of Korea; 6 Department of Family Medicine, Seoul National University Hospital, Seoul, Republic of Korea; 7 Department of Brain and Cognitive Sciences, Seoul National University, Seoul, Republic of Korea; 8 AI Institute, Seoul National University, Seoul, Republic of Korea

**Keywords:** default mode network, executive network, resting-state functional magnetic resonance imaging, salience network, smoking cessation

## Abstract

**Background:**

Tobacco smoking continues to be a leading cause of preventable morbidity and mortality globally, with the success rate of unaided cessation remaining consistently low. Understanding the neurobiological mechanisms of smoking cessation is crucial for improving quit rates. However, there has been a lack of studies examining brain network changes associated with smoking cessation over time. In this study, we aimed to investigate longitudinal changes in the functional connectivity (FC) of large-scale brain networks underlying smoking cessation outcomes using resting-state functional magnetic resonance imaging (fMRI).

**Methods:**

A total of 98 treatment-seeking smokers participated in a 5-week cessation program and underwent resting-state fMRI scans before and after the intervention. Independent component analysis identified the salience network (SN), executive control network (ECN), and default mode network (DMN) components, and region of interest (ROI)-to-ROI FC was compared between successful and unsuccessful quitters using a group × time mixed-effects model. Correlations with smoking-related measures were explored.

**Results:**

Significant group-by-time interaction effects were found in FC, particularly involving connections between SN and ECN, as well as between the SN and DMN. Specifically, successful quitters exhibited greater baseline FC in the SN-ECN and SN-DMN circuits, which tended to decrease and converge toward levels observed in unsuccessful quitters during the cessation process. Exploratory correlational analyses revealed trends suggesting that stronger pre-quit connectivity between the SN and ECN was associated with greater withdrawal severity and longer smoking history in successful quitters.

**Conclusion:**

Taken together, the reduction of initially elevated pre-quit FC in SN-ECN and SN-DMN circuits may reflect an adaptive neural process that supports successful withdrawal management and attentional reallocation during cessation. The identification of these neural substrates not only enhances our mechanistic understanding of smoking cessation over time but also underscores the need for targeted interventions that focus on these neural circuits to enhance quit outcomes.

## 1 Introduction

Tobacco smoking remains a leading cause of preventable morbidity and mortality globally, posing a major public health concern (WHO, 2023). Approximately 70% of smokers express a desire to quit, yet the success rate of unaided cessation hovers below 10%, highlighting a stark disconnect between motivation and the capacity to overcome nicotine dependence (Creamer, 2019). This disconnect underscores the critical need to investigate the underlying neurobiological mechanisms of smoking cessation to develop more effective interventions that can improve quit rates.

Recently, there has been growing interest in understanding the functional connectivity (FC) of large-scale brain networks using resting-state functional magnetic resonance imaging (fMRI). Notably, research has primarily focused on three key networks including the salience network (SN), executive control network (ECN), and default mode network (DMN), as these are considered the most clinically relevant (Menon, 2011, 2018, 2020; Menon et al., 2023). The SN, which includes regions such as the anterior insula and anterior cingulate cortex, is involved in detecting and responding to important stimuli. The ECN, also known as the frontoparietal network, comprises areas such as the dorsolateral prefrontal cortex and posterior parietal cortex, and is implicated in cognitive control, decision-making, and goal-directed behavior. Lastly, the DMN, including regions such as the medial prefrontal cortex, posterior cingulate cortex, and lateral parietal cortex, has been associated with self-referential processing and mind-wandering.

Most previous studies examining large-scale brain networks in smokers have either compared smokers to non-smokers or investigated the effects of abstinent versus satiated states within smokers, but few have specifically addressed smoking cessation outcomes. For example, studies comparing smokers and non-smokers often report decreased FC, notably in the SN (Li et al., 2017; Hobkirk et al., 2018; Yip et al., 2022), ECN (Janes et al., 2012; Weiland et al., 2015; Fedota et al., 2016; Yip et al., 2022), and DMN (Weiland et al., 2015; Claus and Weywadt, 2020), among smokers. Additionally, studies comparing abstinent and satiated states in smokers have shown that FC within and between brain networks vary depending on the smoking state (Lerman et al., 2014; Fedota et al., 2018; Yip et al., 2022). In particular, decreased FC between the SN and ECN, along with increased FC between the SN and DMN, has been identified as a neural signature of smokers during acute abstinence. While these studies have provided valuable insights into the effects of smoking and the impact of nicotine abstinence on brain networks, the relationship between these connectivity alterations and smoking cessation outcomes remains unclear.

Among the limited number of studies examining the association between FC and smoking cessation outcomes, most have relied on a seed-based approach in resting-state fMRI analyses, rather than utilizing network-based methods. These studies have focused on regions such as the insula and striatum, and notable findings highlight the involvement of insula-centered circuits and their connectivity changes related to withdrawal and cessation outcomes (Addicott et al., 2015; Sweitzer et al., 2016; Wang et al., 2020; Wang et al., 2021). While useful for examining regional connectivity changes, seed-based methods may not fully capture the complex interactions across the entire brain network (Smitha et al., 2017). Furthermore, most of the studies investigating resting-state FC with smoking cessation outcomes have only used baseline scans collected before quit attempts to predict later smoking outcomes, limiting the understanding of longitudinal changes in FC.

Taken together, there is a gap in research that has explored the longitudinal changes in large-scale brain network connectivity involved in smoking cessation. Here, the present study aims to address this gap by examining these changes in smokers during a quit attempt. Specifically, we compared smokers who successfully maintained abstinence with those who relapsed during a 6-week cessation program. We hypothesized that successful quitters would exhibit distinct patterns of FC in the SN, ECN, and DMN compared to smokers who relapsed, with successful quitters showing stronger connectivity between the SN and ECN, and weaker connectivity between the SN and DMN at baseline, and these differences may stabilize or diminish as the brain adapts during the cessation process.

## 2 Methods and materials

### 2.1 Participants and procedures

Smokers who reported an intention to quit smoking were recruited through flyers on the university campus and online postings on local community websites. The inclusion criteria for the study were as follows: smoking a minimum of five cigarettes per day for the past year, or an equivalent amount of e-cigarettes as defined by at least five sessions of 15 puffs each or 10 min of smoking at each session (Foulds et al., 2015), and willing to make a quit attempt and enroll in the smoking cessation program at a university health center. Exclusion criteria included major psychiatric or medical illness, use of psychoactive medications, other substance use disorder, including alcohol, as assessed by the Structured Clinical Interview for DSM-5 Disorders (First, 2014), use of illicit drugs based on a urine drug screen (One-Step Drug Abuse Test Dip Card from WHPM, Inc.), and contraindications for magnetic resonance imaging (MRI).

The study protocol involved three laboratory visits: a screening visit, a pre-quit MRI scan visit preceding the smoking cessation program, and a post-quit MRI scan visit upon its completion. Prior to both MRI scans, participants were asked to abstain from smoking, alcohol use, and caffeine consumption for at least 8 h. For traditional cigarette users, abstinence was confirmed by measuring exhaled carbon monoxide (CO) concentrations using a Micro Smokerlyzer (Bedfont Scientific Ltd.), with participants required to exhibit CO levels below 12 ppm following at least 8 h of abstinence (Sandberg et al., 2011). For e-cigarette users, abstinence verification relied on self-report, as vaping does not produce carbon monoxide. Alcohol abstinence was verified by assessing breath alcohol concentration with a breathalyzer (AL8800 ALCOSCAN, Sentech Korea), and caffeine abstinence was confirmed through participant self-report.

The smoking cessation program was 5 weeks with weekly clinic visits and offered both pharmacological and psychosocial treatments. In cases where scheduling was difficult due to holidays, the program was extended to 6 weeks (*n* = 19, 20.9%). Consistent with previous studies, smoking abstinence was defined as continuous abstinence over the last 4 weeks of the program (Gonzales et al., 2006; Jorenby et al., 2006). Participants who remained abstinent during this period were classified as successful quitters, and those who did not maintain abstinence as unsuccessful quitters, based on self-report and cross-validation by CO levels of 5 ppm or below for traditional cigarette users (Perkins et al., 2013). For e-cigarette users, abstinence verification relied on self-report only. The study procedures were approved by the Institutional Review Boards of Seoul National University and were performed in accordance with the ethical standards of the Declaration of Helsinki. Written consent form was obtained from all participants to confirm their voluntary participation.

### 2.2 Clinical assessments

Demographic information and self-report measures including smoking-related assessments were obtained at the screening visit after confirming eligibility. Nicotine dependence was assessed with the Fagerström Test of Nicotine Dependence (FTND) (Heatherton et al., 1991; Ahn et al., 2002) and Cigarette Dependence Scale (CDS-12) (Etter, 2005a). Nicotine craving was evaluated with the Brief Questionnaire on Smoking Urges (QSU-Brief) (Cox et al., 2001). Nicotine withdrawal was assessed with the Cigarette Withdrawal Scale (CWS) (Etter, 2005b) and Minnesota Nicotine Withdrawal Scale (MNWS) (Hughes and Hatsukami, 1986; Kim et al., 2007). Daily cigarette consumption was initially recorded as a categorical variable as follows: 0 for none, 1 for 1–4 cigarettes, 2 for 5–10 cigarettes, 3 for 11–20 cigarettes, and 4 for greater than 20 cigarettes per day. During the latter two-thirds of the study period, we began collecting the daily cigarette consumption data as a continuous variable, detailing the exact number of cigarettes smoked per day. For the 20% of our sample who had only provided categorical responses, we inferred daily averages by assigning median values to each category: 0 equating to 0 cigarettes; 1 to 3 cigarettes (median of 1 and 4); 2 to 7.5 cigarettes (median of 5 and 10); 3 to 15.5 cigarettes (median of 11 and 20); and 4 to 30 cigarettes (median of 20 and 40). For electronic cigarettes, 15 puffs or 10 min of smoking were considered equivalent to one session, comparable to one regular cigarette (Foulds et al., 2015). Duration of smoking was calculated by subtracting the age of smoking initiation from the current age.

### 2.3 MRI data acquisition and preprocessing

All MRI scanning was conducted on a Siemens TIM Trio 3 T scanner using a 32-channel head coil. A high-resolution T1-weighted structural image was acquired using an MPRAGE sequence (TR = 2,300 ms, TE = 2.36 ms, field of view = 256 mm, flip angle = 9°, slices = 224, and slice thickness = 1 mm). Resting-state fMRI was performed using a T2^*^-weighted, single-shot echo, echo-planar imaging sequence (TR = 1,500 ms, TE = 30 ms, field of view = 256 mm, flip angle = 85°, slices = 64, slice thickness = 2.3 mm, and 250 volumes for a total duration of 6 min 44 s). During the resting-state fMRI scan, participants were instructed to rest with their eyes open while fixating on a cross in the center of the screen. An infrared camera attached to the head coil was used to monitor alertness. These images were collected as part of a 60-min scanning session in the following order: a localizer, T1-weighted structural MRI, resting-state fMRI, task-based fMRI, and neuromelanin-sensitive MRI.

### 2.4 Data preprocessing and analysis

Resting-state fMRI analyses consisted of (1) preprocessing, (2) independent component analysis (ICA), (3) first-level calculation of resting-state FC matrices of the ROIs based on the ICA results, (4) second-level group (successful quit group vs. unsuccessful quit group) x time (pre and post) interaction analyses controlling for age, gender, and smoking duration, (5) and correlation analyses with self-report measures. Preprocessing and analyses of the resting-sate fMRI data were performed using the CONN toolbox (RRID: SCR_009550) release 21.a (Whitfield-Gabrieli and Nieto-Castanon, 2012; Nieto-Castanon and Whitfield-Gabrieli, 2021) with SPM12.

#### 2.4.1 Preprocessing

Functional and anatomical data were preprocessed using a standard preprocessing pipeline including realignment, slice timing correction, outlier detection, direct segmentation, MNI-space normalization, and smoothing. Functional data alignment was performed using the SPM realign & unwarp procedure, with all scans coregistered to the first scan of the initial session via a least squares method and a 6-parameter (rigid body) transformation. B-spline interpolation was applied to correct for motion and magnetic susceptibility interactions, and temporal misalignment between different slices of the functional data (acquired in interleaved Siemens order) was corrected following SPM slice-timing correction procedure. Potential outlier scans were identified using ART as acquisitions with framewise displacement above 0.5 mm or global BOLD signal changes above 3 standard deviations, and a reference BOLD image was computed for each subject by averaging all scans excluding outliers. Functional and anatomical data were then normalized into standard MNI space, segmented into grey matter, white matter, and CSF tissue classes, and resampled to 2 mm isotropic voxels. Lastly, functional data were smoothed using spatial convolution with a Gaussian kernel of 8 mm full width half maximum (FWHM). In addition, functional data were denoised using a standard denoising pipeline, which involved regressing out potential confounding effects. These included white matter timeseries (5 CompCor noise components), CSF timeseries (5 CompCor noise components), motion parameters and their first order derivatives (12 factors), outlier scans (below 58 factors), session and task effects and their first order derivatives (4 factors), and linear trends (2 factors) within each functional run. Outlier scans (below 58 factors) refer to the maximum number of outlier timeframe regressors that can be included in the denoising model while maintaining adequate degrees of freedom for reliable connectivity estimation. The BOLD timeseries was then bandpass filtered between 0.008 Hz and 0.09 Hz.

#### 2.4.2 Group independent component analysis

Group-level independent component analyses (group-ICA) were performed to estimate 20 temporally coherent networks from the resting-state fMRI data across all participants and sessions (Calhoun et al., 2001). The BOLD signal from every timepoint and voxel in the brain was concatenated across participants and sessions along the temporal dimension. A singular value decomposition of the z-score normalized BOLD signal with 64 components separately for each participant and session was used as a subject-specific dimensionality reduction step. The dimensionality of the concatenated data was further reduced using a group-level singular value decomposition with 20 components, and a fast-ICA fixed-point algorithm with hyperbolic tangent (G1) contrast function was used to identify spatially independent group-level networks from the resulting components. Lastly, GICA3 back-projection was used to compute ICA maps associated with these same networks separately for each participant and session. Each ICA map was visually checked for the SN, ECN, and DMN networks (Menon, 2011) and cross-validated with the external template provided in the CONN toolbox. The ICA maps were thresholded at Z ≥ 3.0 for the group-level connectivity analysis.

#### 2.4.3 First-level and second-level network analyses

All clusters within the SN, ECN, DMN maps were selected as regions of interests (ROIs). In the first-level analysis, ROI-to-ROI connectivity matrices were estimated characterizing the FC between each pair of regions among 21 ROIs, resulting in a total of 210 (=21x (21–1) / 2) connections. For each subject, the mean BOLD time-series was extracted from all voxels within each ROI cluster. ROI-to-ROI functional connectivity was then computed as Fisher-transformed bivariate correlation coefficients between the extracted time-series of each ROI pair. For each ROI-to-ROI connection, we estimated a general linear model with the following structure: FC (connection, subject, time) = *β*₀ + *β*₁(Group) + *β*₂(Time) + *β*₃(Group×Time) + *β*₄(Age) + *β*₅(Sex) + *β*₆(Smoking_Duration) + *ε*, where group (successful quit group vs. unsuccessful quit group) and time (pre vs. post) were treated as fixed effects, and age, sex, and smoking duration were included as covariates. The model accounted for repeated measurements within subjects. Connection-level hypotheses were evaluated using multivariate parametric statistics with random-effects analysis across subjects. Cluster-level inference was performed using multivariate parametric statistics (MVPA omnibus test), which tests whether a group of connections shows a significant effect collectively.

Significance was determined at the level of clusters (groups of connections). Connection-level hypotheses were evaluated using multivariate parametric statistics with random-effects across participants and sample covariance estimation across pre-quit and post-quit measurements. All analyses were conducted at the ROI-to-ROI level (210 connections), examining connections within and between the three networks of interest, not aggregate network-level measures. Inferences were performed at the level of individual clusters, where clusters represent groups of connections that are similar based on anatomical proximity and functional similarity. Cluster-level inferences were based on parametric statistics using functional network connectivity (Jafri et al., 2008), with connections grouped using a complete-linkage hierarchical clustering procedure based on ROI-to-ROI anatomical proximity and functional similarity metrics. Results were corrected for multiple comparisons using FDR across all identified clusters. The statistical significance level was set at a connection-level threshold of *p* < 0.05 (uncorrected) and a cluster-level threshold of p-FDR < 0.05.

#### 2.4.4 Correlational analyses between network-based resting-state FC and self-report measures

Statistical analyses of the demographic and self-report data were conducted using R software (version 4.3.2). Baseline characteristics of groups were compared using independent t-test for continuous variables, and chi-square test for categorical variables. Subsequently, we performed correlational analysis to explore the relationships between FC values and self-report measures including smoking-related variables. To do this, FC values of the connections showing significant group × time interaction effects were extracted for both pre-quit and post-quit timepoints and correlated with smoking-related variables using Pearson’s correlation coefficients.

## 3 Results

### 3.1 Demographic and smoking-related characteristics

Of 98 participants who had both pre- and post-quit resting-state fMRI scans, 6 participants were excluded due to using different MRI acquisition parameters, and 1 participant was excluded for prematurely attempting to quit before the smoking cessation program started. Consequently, 91 participants were included in the final analysis. Demographic and smoking-related characteristics for the unsuccessful and successful quit groups are summarized in Table 1. According to the smoking cessation criterion defined in our study, which is complete abstinence during the final 4 weeks, 26 participants were classified as successful quitters, and 65 participants were classified as unsuccessful quitters. At the pre-quit visit, successful quitters did not differ significantly from unsuccessful quitters in terms of age, sex, education, daily smoking amount, CO level, or the severity of nicotine dependence as measured by the FTND scores. Successful quitters had a longer smoking duration (*p* = 0.006) than unsuccessful quitters at the pre-quit visit. At the post-quit visit, successful quitters had higher program participation rates (*p* = 0.003) and lower CO levels (*p* = 0.001) than unsuccessful quitters.

**Table 1:** Demographic and smoking-related characteristics.

Characteristic	Unsuccessful quitters (*n* = 65)	Successful quitters (*n* = 26)	Test Statistic	*p*-value
Age, years	25.35 ± 3.99	26.23 ± 3.37	−0.97	0.3
Sex, male:female	55:10	22:4	0.00	1.0
Education, year	16.12 ± 2.25	16.54 ± 2.93	−0.73	0.5
Smoking amount, cigarette/day	11.67 ± 6.92	9.50 ± 4.01	1.5	0.14
Smoking duration, year	4.57 ± 4.15	7.31 ± 4.25	−2.8	0.006**
FTND score	2.54 ± 1.75	1.92 ± 1.55	1.6	0.12
Program participation, %	53.08 ± 31.99	74.36 ± 22.72	−3.1	0.003**
CO level at pre-quit, ppm	4.80 ± 4.60	4.00 ± 4.14	0.77	0.4
CO level at post-quit, ppm	4.46 ± 3.86	1.85 ± 1.05	3.4	0.001**

Values are presented as mean ± standard deviation, unless otherwise indicated.

**p < 0.01; *p < 0.05.

CO, Carbon Monoxide; FTND, Fagerström Test of Nicotine Dependence.

Smoking type and treatment information are summarized in Table 2. The majority of participants (81.3%) smoked traditional cigarettes only, while 8.8% used e-cigarettes only, and 9.9% used both. There was no significant group difference in smoking type (*χ*^2^ = 4.14, *p* = 0.126). Notably, there were no dual users (cigarettes + e-cigarettes) in the successful quit group (0%), whereas 13.8% of unsuccessful quitters were dual users. All participants received psychosocial treatment, and 67% additionally received pharmacological treatment (nicotine replacement therapy and/or varenicline). A higher proportion of successful quitters (80.8%) received combined psychosocial and pharmacological treatment compared to unsuccessful quitters (61.5%), though this difference was not statistically significant (*χ*^2^ = 2.30, *p* = 0.129). Detailed treatment information by participant is provided in Supplementary Table S1.

**Table 2:** Treatment and smoking characteristics by group.

Characteristic	Unsuccessful quitters (*n* = 65)	Successful quitters (*n* = 26)	Overall (*n* = 91)	*p*-value
Smoking type				0.126
Cig only	51 (78.5%)	23 (88.5%)	74 (81.3%)	
E-Cig only	5 (7.7%)	3 (11.5%)	8 (8.8%)	
Both	9 (13.8%)	0 (0%)	9 (9.9%)	
Treatment type: 2 categories				0.129
Psychosocial only	25 (38.5%)	5 (19.2%)	30 (33%)	
Psychosocial +	40 (61.5%)	21 (80.8%)	61 (67%)	
Pharm and/or NRT				
Treatment type: 4 categories				0.357
Psychosocial only	25 (38.5%)	5 (19.2%)	30 (33%)	
Psychosocial + Pharm	30 (46.2%)	15 (57.5%)	45 (49.5%)	
Psychosocial + NRT	7 (10.8%)	4 (15.4%)	11 (12.1%)	
Psychosocial + Both	3 (4.6%)	2 (7.7%)	5 (5.5%)	

Values are presented as *n* (%). *p*-values were calculated using the Chi-square test.

NRT, Nicotine Replacement Therapy; Pharm, Pharmacological Treatment.

### 3.2 Head motion and data quality

To assess potential confounds related to head motion, we compared mean framewise displacement (FD) and the number of scrubbed volumes between groups at both timepoints. There were no significant group differences in mean FD or scrubbed volumes at either pre- or post-quit scans (see Supplementary Table S2 for details).

### 3.3 Group ICA

Of the 20 group-ICA components, 5 components were identified as the SN, ECN, and DMN:

1. the SN, including the bilateral superior frontal gyrus, middle frontal gyrus, dorsal anterior cingulate cortex (ACC)/paracingulate gyrus, anterior insula, right cerebellum, and left precuneus (Supplementary Figure S1A),
2. the left ECN, including the left dorsolateral prefrontal cortex (DLPFC) and angular gyrus/inferior parietal gyrus (Supplementary Figure S1B) and right ECN, including the right DLPFC, angular gyrus/inferior parietal gyrus, orbital frontal gyrus, inferior temporal gyrus, and cerebellum (Supplementary Figure S1C),
3. the DMN, including the left medial PFC, angular gyrus, and inferior temporal gyrus (Supplementary Figure S1D), and PCC (Supplementary Figure S1E).

Spatial cross-correlation of these networks with the external template revealed a mean r of 0.34 with a range between 0.20 and 0.44. A total of 21 ROIs were derived from five ICA components (SN, left ECN, right ECN, and two DMN). Detailed information on all 21 ROIs, including component number, parent network, anatomical label, peak MNI coordinates, and cluster size, is provided in Supplementary Table S3.

### 3.4 Network analysis

**Figure 1:**
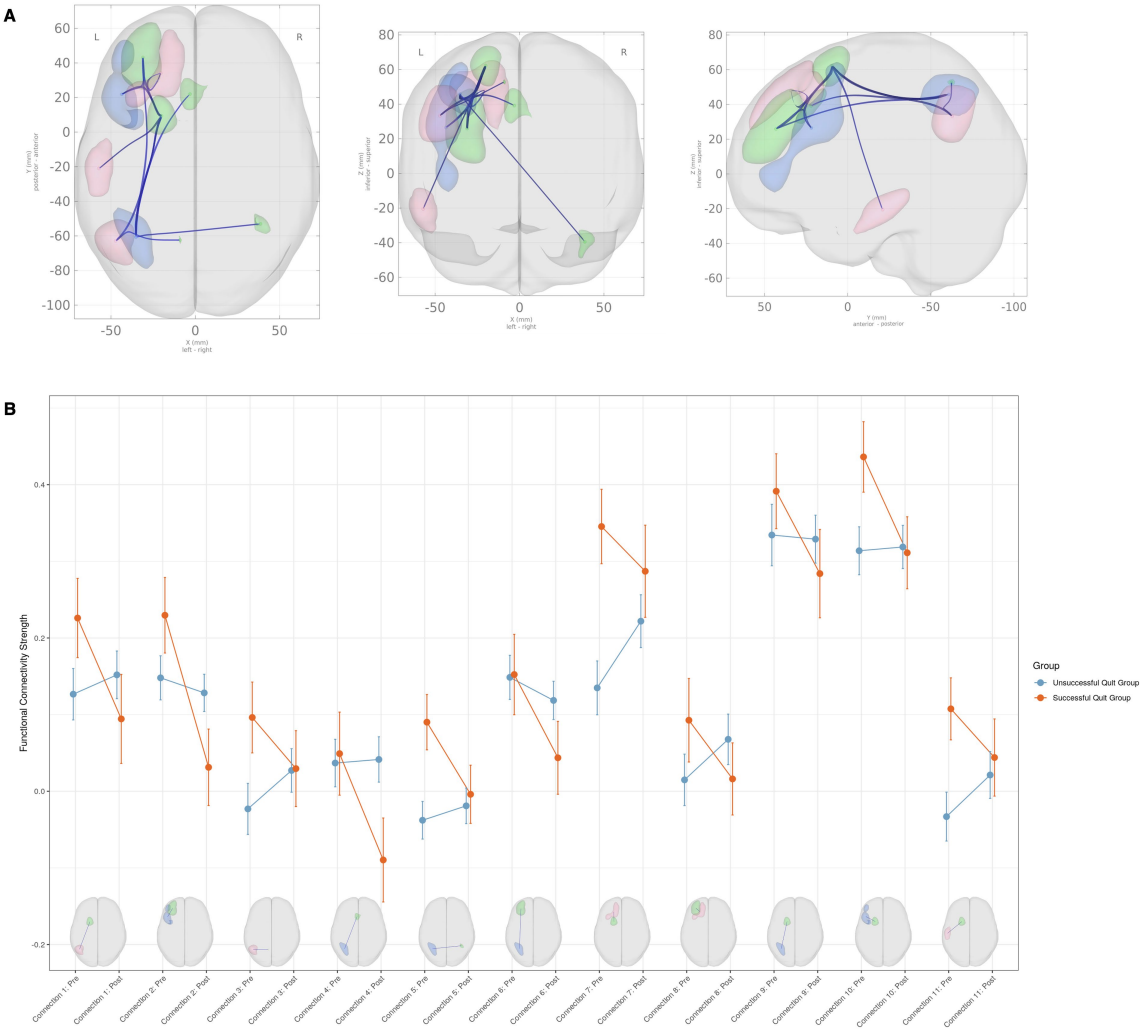
Group × time interaction effects on functional connectivity among large-scale brain networks. **(A)** Brain regions showing significant group (successful quit group vs. unsuccessful quit group) × time (pre-quit vs. post-quit) interaction effects. The clusters belong to the Salience Network (green), Default Mode Network (red), and Executive Control Network (blue). **(B)** Interaction plots displaying connectivity strength (Fisher Z-transformed correlation coefficients) for each significant connection. Error bars represent the standard error of the mean. Please refer to Table 3 for detailed information on the anatomical regions.

As shown in Figure 1, significant group-by-time interaction effects on FC were found across 11 inter-network connections, consisting of 5 SN-DMN and 6 SN-EN connections (*F*(3, 84) = 5.54, p-uncorrected = 0.0016, p-FDR = 0.0097). Details about the peak MNI coordinates and their corresponding anatomical regions for each significant connection are summarized in Table 3. No significant group differences were observed at either the pre- or post-quit visits.

**Table 3:** Regions with significant group (successful quit group × unsuccessful quit group) by time (pre × post) interaction on functional connectivity.

Connection number	Network	Anatomical region	Peak MNI coordinates		*T* value
1	SN–DMN	L. SFG–L. Angular gyrus	−20, 9, 62	−47, −63, 33	−2.83
2	SN–ECN	L. MFG–L. DLPFC	−31, 43, 25	−44, 22, 27	−2.74
3	SN–DMN	L. Precuneus–L. Angular gyrus	−9, −62, 53	−47, −63, 33	−2.45
4	SN–ECN	L. ACC–L. IPG	−3, 22, 39	−35, −61, 46	−2.54
5	SN–ECN	R. Cerebellum–L. IPG	39, −53, −40	−35, −61, 46	−2.26
6	SN–ECN	L. MFG–L. IPG	−31, 43, 26	−35, −61, 46	−2.14
7	SN–DMN	L. SFG–L. SFG	−20, 9, 62	−20, 34, 48	−2.44
8	SN–DMN	L. MFG–L. SFG	−31, 43, 26	−20, 34, 48	−2.29
9	SN–ECN	L. SFG–L. IPG	−20, 9, 62	−35, −61, 46	−1.99
10	SN–ECN	L. SFG–L. DLPFC	−20, 9, 62	−44, 22, 27	−2.07
11	SN–DMN	L. SFG–L. ITG	−20, 9, 62	−57, −21, −21	−2.07

*T* values represent the group (successful vs. unsuccessful quitters) × time (pre-quit vs. post-quit) interaction effect. Negative *T* values indicate that successful quitters showed higher functional connectivity at pre-quit compared to unsuccessful quitters, with this difference diminishing by post-quit (i.e., successful quitters exhibited a greater decrease in connectivity over time relative to unsuccessful quitters).

ACC, Anterior Cingulate Cortex; DLPFC, Dorsolateral Prefrontal Cortex; DMN, Default Mode Network; ECN, Executive Control Network; IPG, Inferior Parietal Gyrus; ITG, Inferior Temporal Gyrus; L, Left; MFG, Middle Frontal Gyrus; MNI, Montreal Neurological Institute; R, Right; SN, Salience Network; SFG, Superior Frontal Gyrus.

Among the SN-DMN connections, the successful smoking cessation group generally exhibited greater connectivity compared to the unsuccessful group at the pre-quit visit, with these differences diminishing by the post-quit visit (Figure 1B). A similar pattern was observed in the SN-ECN connections, where FC in the successful smoking cessation group was higher at the pre-quit visit and stabilized by the post-quit visit (Figure 1B). A few exceptions were noted within the SN-ECN network. Specifically, for connections between SN (left middle frontal gyrus) and ECN (left DLPFC) (connection #2), between SN (left ACC) and ECN (left inferior parietal gyrus) (connection #4), and between SN (left middle frontal gyrus) and ECN (inferior parietal gyrus) (connection #6), connectivity in the successful cessation group was comparable to the unsuccessful group at the pre-quit visit but decreased at the post-quit visit, falling below levels observed in the unsuccessful group (Figure 1B; Table 3).

### 3.5 Relationship to smoking-related measures

Correlations were calculated between the FC values from each significant connection and smoking-related variables, resulting in 77 tests (11 FC values x 7 smoking-related variables). Of note, only pre-quit visit correlations were analyzed, as the smoking-related questionnaires were administered only at the pre-quit visit and not at the post-quit visit. A Bonferroni correction was applied to account for multiple comparisons with a significance threshold set at *p* < 0.05/77 (*p* < 0.0006). After applying this correction, none of the correlations reached statistical significance. However, several trends emerged with uncorrected *p* < 0.05, suggesting potential relationships that warrant further investigation.

**Figure 2:**
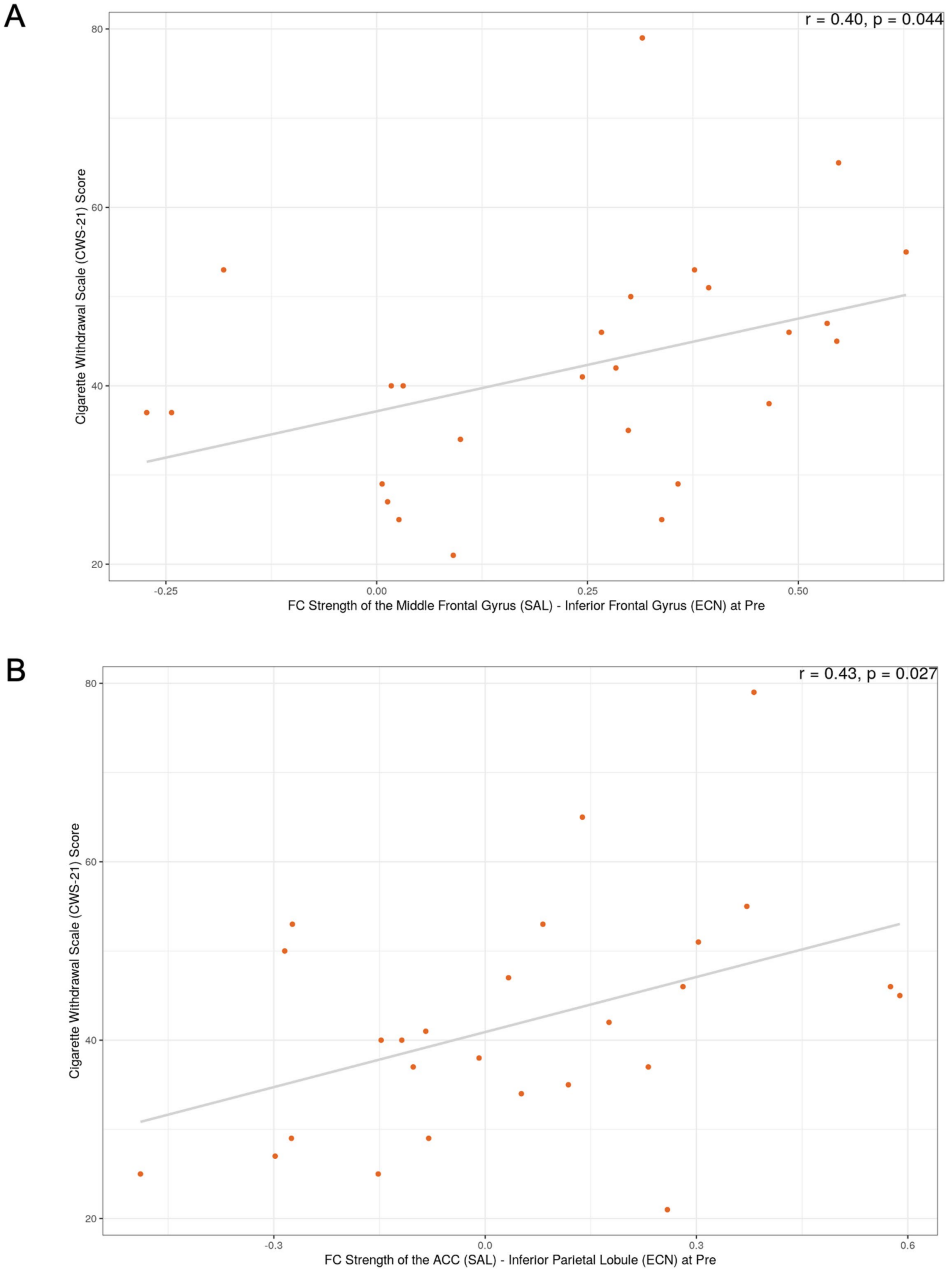
Exploratory correlations between functional connectivity and withdrawal severity in the successful smoking cessation group. **(A)** Correlation between FC strength of the SN (middle frontal gyrus)–ECN (DLPFC) at the pre-quit visit and the Cigarette Withdrawal Scale (CWS) score. **(B)** Correlation between FC strength of the SN (ACC)–ECN (inferior parietal cortex) at the pre-quit visit and the CWS score. These exploratory analyses did not survive correction for multiple comparisons; *p*-values shown are uncorrected.

The most notable correlation findings were observed in the SN-ECN connections of the successful smoking cessation group. For the FC strength between the SN (middle frontal gyrus) and ECN (DLPFC) at the pre-quit visit (connection #2), a positive correlation was found with nicotine withdrawal severity, as measured by the CWS (*r* = 0.4, uncorrected *p* = 0.04; Figure 2A), as well as with smoking duration (*r* = 0.49, uncorrected *p* < 0.05). Additionally, for the FC strength between the SN (ACC) and ECN (inferior parietal cortex) at the pre-quit visit (connection #4), a positive correlation with nicotine withdrawal severity was observed in the successful quit group (*r* = 0.43, uncorrected *p* = 0.027; Figure 2B). In the unsuccessful quit group, weak associations were also found with nicotine dependence severity, as measured by the FTND (*r* = 0.28, uncorrected *p* < 0.05) and CDS (*r* = 0.32, uncorrected *p* < 0.05) for the same connection. Furthermore, significant positive correlations were identified between three other SN-ECN connections (connections #5, #9, and #10) and smoking duration. The complete correlation results are shown in Supplementary Figures S2, S3.

## 4 Discussion

The present study investigated the dynamic changes in FC within and between the large-scale brain networks during a 6-week smoking cessation period, specifically focusing on the SN, ECN, and DMN, and comparing these patterns between successful quitters and unsuccessful quitters. Our network analysis revealed significant group-by-time interaction effects on FC, driven by changes in inter-network connectivity between the SN–DMN and SN–ECN. A key finding was observed in the SN–DMN connections: the successful quit group exhibited relatively higher FC in these pathways at the pre-quit visit compared to the unsuccessful quit group, with these differences largely diminishing by the post-quit visit. Similarly, the SN–ECN connections in the successful quit group showed higher FC at the pre-quit visit relative to the unsuccessful quit group, with most of these connections stabilizing at comparable levels by the post-quit visit. A few exceptions were observed in the SN-ECN connections, where FC, initially comparable between the groups at the pre-quit visit, declined in the successful cessation group at the post-quit visit.

The observed group-by-time interactions offer several interpretative possibilities. The SN is known to mediate the detection of salient stimuli and to facilitate dynamic switching between the internally directed DMN and the externally focused ECN (Menon and Uddin, 2010; Uddin, 2015). Neuroimaging studies have shown that during cognitively demanding tasks, FC between the SN and ECN is enhanced to allocate attention to external stimuli, while FC between the SN and DMN is decreased to reduce internal self-referential processing (Schimmelpfennig et al., 2023). In a similar vein, prior neuroimaging research in addiction shows that during withdrawal and craving states, this pattern is disrupted, with a reduction in connectivity between the SN and ECN and increased connectivity between the SN and DMN, reflecting a shift toward internally focused attention and impaired cognitive control (Fedota and Stein, 2015). This shift is thought to destabilize cognitive control, promote internally directed attention, and exacerbate craving symptoms, thereby increasing relapse vulnerability. Based on this, we hypothesized that successful quitters would show a decrease in SN-DMN connectivity and an increase in SN-ECN connectivity. However, unexpectedly, we observed an increase in both SN-DMN and SN-ECN connectivity at the pre-quit visit, which decreased and converged toward levels observed in unsuccessful quitters by the post-quit visit, with a few exceptions.

Accumulating evidence challenges the traditional view, suggesting that network interactions among the SN, DMN, and ECN dynamically change depending on task goals and demands (Geng et al., 2024). Notably, some studies have shown that in working memory tasks with increasing cognitive load, the SN exhibits increased connectivity with the DMN, in addition to its enhanced coupling with the ECN (Liang et al., 2016; Finc et al., 2017). These findings imply that the modulation between these networks is not always unidirectional; instead, under certain conditions, there may be an initial increase in SN-DMN connectivity, reflecting an attempt to integrate or monitor internally generated signals. In this context, the higher pre-quit connectivity observed in the successful quit group in both SN-DMN and SN-ECN connections may reflect an adaptive allocation of cognitive resources and interoceptive awareness, enabling individuals to more effectively manage cessation-induced symptoms. Furthermore, the convergence of these connectivity patterns may imply a return to baseline levels following successful cessation.

Our correlational analysis of FC metrics and smoking-related measures may further help interpret the results. Although none of these relationships reached significance after correcting for multiple comparisons, FC between the SN (middle frontal gyrus) and ECN (DLPFC) showed moderate positive correlations with both withdrawal severity and smoking duration in the successful quit group. Similarly, FC between the SN (ACC) and ECN (inferior parietal cortex) was positively related to withdrawal severity in the successful quit group. These findings suggest that higher baseline FC within SN–ECN circuits may be associated with greater withdrawal symptoms and longer smoking histories. Interestingly, these two connections were among the exceptions where baseline FC showed no group differences, but later decreased in successful quitters compared to unsuccessful quitters. One possible interpretation of the decrease is a downregulation of overactive salience signals, which may support the reestablishment of a balanced neural state. This reduction in hypervigilance and excessive attentional bias toward withdrawal-related cues, factors frequently implicated in relapse, could ultimately facilitate successful cessation (Deshpande et al., 2022).

Our findings contribute to the growing body of literature suggesting that the functional interplay between large-scale brain networks—particularly among the SN, DMN, and ECN—plays a pivotal role in nicotine addiction and smoking cessation outcomes (Lerman et al., 2014; Yip et al., 2022). In successful quitters, the higher pre-quit FC within SN–DMN and SN–ECN connections might reflect an initially heightened state of neural communication geared toward processing interoceptive and salient information. Such a state, however, appears to diminish once smoking cessation is achieved, potentially indicating a neural recovery or reorganization process that underpins successful behavior change.

It is also noteworthy that no significant group differences were identified at the individual pre- and post-quit visits; rather, the significant effects emerged only in the group-by-time interaction analysis. This pattern suggests that it is not the absolute level of connectivity at a given moment that matters, but rather the dynamic changes in connectivity over time that are most predictive of cessation outcomes. This dynamic perspective is consistent with recent data-driven connectivity approaches that emphasize the importance of capturing both shared and unique individual-level network alterations over the course of treatment (Murray et al., 2024).

This study has several limitations that should be considered when interpreting the findings. First, our sample was predominantly young (mean age ~25 years) and male (84.6%), recruited from a single site, which may limit the generalizability of our findings to broader populations, including older adults, women, and individuals from different cultural backgrounds. Second, although e-cigarette users represented a small proportion of our sample (8.8% exclusive e-cigarette users; 9.9% dual users), their inclusion presents methodological challenges: not only may nicotine consumption patterns vary substantially depending on device type and e-liquid concentration, but also biochemical verification of abstinence using CO levels is not valid for e-cigarette users, requiring reliance on self-report, which may be subject to bias. Third, the spatial correlation between our ICA-derived networks and external templates was modest (mean r = 0.34, range 0.20–0.44), suggesting that our network definitions may not perfectly correspond to canonical network boundaries. Additionally, the anterior DMN component appeared left-lateralized after thresholding (*Z* ≥ 3.0), which may have limited our ability to detect certain connectivity patterns involving right-lateralized DMN regions. Fourth, the absence of a never-smoker control group limits our ability to determine whether the observed connectivity changes represent true normalization or an adaptive recalibration specific to the cessation process. Finally, exploratory correlational analyses did not survive correction for multiple comparisons and should be considered hypothesis-generating. Future studies with larger, more diverse samples and never-smoker controls are needed to validate and extend these findings.

In summary, our findings suggest that successful smoking cessation is associated with distinct alterations in FC across key brain networks involved in attention, salience, and cognitive control. In particular, a higher pre-quit FC in SN–DMN and SN–ECN connections, which is selectively modulated during abstinence, appears to be an important marker of cessation success. Furthermore, the observed trends linking SN–ECN connectivity to nicotine withdrawal severity and smoking duration highlight the potential clinical relevance of these network measures as biomarkers of treatment response. Although these relationships did not survive very conservative statistical correction, they provide a compelling impetus for further investigation. Future studies are needed to replicate and extend these findings, ideally incorporating larger sample sizes, longitudinal designs, and complementary analytic techniques such as dynamic FC and graph theory approaches. Such work could further clarify the role of network-level connectivity alterations as predictors of smoking cessation outcomes and identify novel targets for neuromodulation or pharmacological intervention.

Overall, this study contributes to a more nuanced understanding of the interplay between large-scale brain networks in the context of smoking cessation. By focusing on changes in inter-network connectivity over time, our findings underscore the importance of dynamic neural reorganization during the cessation process. Such longitudinal changes, as reflected in the convergence of SN–DMN and SN–ECN interactions, may represent a key neurobiological mechanism that supports successful abstinence. The identification of these network markers has significant implications for developing targeted, personalized interventions that address both the cognitive and affective dimensions of nicotine withdrawal and addiction.

## 5 Conclusion

In conclusion, this study provides novel longitudinal evidence that successful smoking cessation is accompanied by dynamic reorganization in large-scale brain networks, particularly involving the salience, executive control, and default mode networks. Successful quitters displayed heightened pre-quit connectivity between the SN–ECN and SN–DMN circuits, which subsequently decreased following cessation, suggesting that adaptive modulation of these networks may facilitate withdrawal management and attentional control during abstinence. These results highlight the importance of considering brain network dynamics rather than static connectivity patterns in understanding the neural mechanisms underlying behavior change. By revealing that the reduction of initially elevated network coupling may serve as a neural marker of successful cessation, our findings underscore the potential for developing targeted neurobiological interventions such as neuromodulation or cognitive training that enhance these adaptive processes. Future research incorporating larger samples and dynamic connectivity analyses is warranted to validate these findings and to clarify how time-dependent neural reconfiguration supports long-term abstinence from smoking.

## Data Availability

The data analyzed in this study is subject to the following licenses/restrictions: de-identified data are available from the corresponding author upon reasonable request and subject to institutional approvals. Requests to access these datasets should be directed to W-YA, wahn55@snu.ac.kr.
