# Supplementary material for: Longitudinal brain connectivity changes associated with successful smoking cessation
Source: Front Psychol. 2025 Dec 17;16:1734803. doi: 10.3389/fpsyg.2025.1734803 (PMC12753871; doi:10.3389/fpsyg.2025.1734803)
Supplement: Supplementary file 1 [file Data_Sheet_1.pdf]

Supplementary Information

Supplementary Table 1. Treatment Type by Participant

Participant ID	Group	Pharm	NRT	Pharm Specified	NRT Specified
SMO19301	0	0	1		Patch
SMO19303	0	1	1	Varenicline	Gum
SMO19304	0	0	0		
SMO19305	0	0	0		
SMO19306	0	0	0		
SMO19308	0	0	1		Patch, Gum
SMO19403	1	0	1		Gum
SMO19404	1	0	1		Gum
SMO19405	0	0	1		Gum
SMO19406	0	0	0		
SMO19407	0	1	0	Varenicline	
SMO19410	0	1	0	Varenicline	
SMO19413	0	0	0		
SMO19414	1	1	0	Varenicline	
SMO19415	0	0	0		
SMO19502	0	0	1		Patch, Gum, Candy
SMO19505	0	0	0		
SMO19506	1	0	0		
SMO19507	0	0	0		
SMO21101	0	0	0		
SMO21102	0	1	0	Varenicline	
SMO21103	0	1	0	Varenicline	
SMO21104	0	1	0	Varenicline	
SMO21106	0	1	0	Varenicline	
SMO21203	0	1	0	Varenicline	
SMO21204	0	1	0	Varenicline	
SMO21205	1	1	0	Varenicline	
SMO21301	1	0	1		Patch
SMO21302	0	1	0	Varenicline	
SMO21303	1	1	0	Varenicline	
SMO21304	1	0	0		
SMO21401	0	0	0		
SMO21402	0	1	0	Varenicline	
SMO21403	1	1	0	Varenicline	
SMO21405	0	1	0	Varenicline	
SMO21406	1	0	1		Gum
SMO21407	1	1	0	Varenicline	
SMO21408	0	0	0		
SMO21409	0	1	0	Varenicline	
SMO21503	0	1	0	Varenicline	
SMO21504	0	0	0		
SMO21505	0	1	1	Varenicline	Patch, Gum
SMO21506	1	1	0	Varenicline	
SMO22001	0	1	0	Varenicline	
SMO22003	0	1	0	Varenicline	
SMO22101	0	0	0		
SMO22103	0	0	0		
SMO22104	0	1	0	Varenicline	

SMO22106	0	1	0	Varenicline	
SMO22107	0	1	0	Varenicline	
SMO22108	0	0	0		
SMO22109	0	1	0	Varenicline	
SMO22110	0	1	0	Varenicline	
SMO22201	0	1	0	Varenicline	
SMO22202	1	1	0	Varenicline	
SMO22203	0	0	0		
SMO22204	0	0	0		
SMO22205	0	0	0		
SMO22206	0	1	0	Varenicline	
SMO22207	1	1	0	Varenicline	
SMO22208	0	1	0	Varenicline	
SMO22210	0	1	0	Varenicline	
SMO22211	0	1	0	Varenicline	
SMO22301	0	0	0		
SMO22302	0	1	1	Varenicline	Gum
SMO22303	0	0	0		
SMO22304	1	0	0		
SMO22305	1	1	0	Varenicline	
SMO22306	1	0	0		
SMO22307	1	1	0	Varenicline	
SMO22401	1	1	0	Varenicline	
SMO22403	1	1	1	Varenicline	Gum
SMO22404	0	0	0		
SMO22405	0	0	0		
SMO22406	0	0	0		
SMO22407	0	0	1		Gum
SMO22408	0	0	0		
SMO22409	0	0	1		Gum
SMO22501	1	1	0	Varenicline	
SMO22503	0	1	0	Varenicline	
SMO22505	0	1	0	Varenicline	
SMO22506	0	1	0	Varenicline	
SMO22507	1	1	0	Varenicline	
SMO22508	0	0	0		
SMO22510	1	0	0		
SMO22511	0	1	0	Varenicline	
SMO23001	1	1	0	Varenicline	
SMO23003	0	0	1		Gum
SMO23004	0	1	0	Varenicline	
SMO23005	1	1	0	Varenicline	
SMO23006	1	1	1	Varenicline	Gum

Group is coded as 0 for unsuccessful quitters and 1 for successful quitters.

Abbreviations: NRT: Nicotine Replacement Therapy; Pharm: Pharmacological Treatment.

Supplementary Table 2. Head Motion and Scrubbed Volumes by Group

Variable	Unsuccessful Quit Group	Successful Quit Group	Test Statistic	*p*-value
FD (mm) (Pre-quit session)	1.70 (1.24)	1.58 (1.18)	0.672	0.718
FD (mm) (Post-quit session)	1.62 (1.07)	1.44 (0.94)	0.468	0.480
Scrubbing (Pre-quit session)	7.69 (7.15)	9.58 (10.21)	0.320	0.781
Scrubbing (Post-quit session)	11.46 (11.79)	11.74 (12.41)	0.921	0.954

Values are presented as mean (SD). Scrubbing represents the number of volumes removed due to excessive motion. Group comparisons were conducted using Welch's t-tests.

Abbreviations: FD: Framewise Displacement (mm).

Supplementary Table 3. Anatomical Definitions of 21 ROIs Extracted from Five ICA Components

ICA Number	Parent Network	Anatomical Label	Peak MNI Coordinates	Cluster size (voxels)
ICA 4	SN	L. Middle frontal gyrus	-31, 43, 26	2449
ICA 4	SN	R. Middle frontal gyrus	30, 44, 30	856
ICA 4	SN	L. Superior frontal gyrus	-20, 9, 62	806
ICA 4	SN	L. Anterior cingulate cortex	-3, 22, 39	360
ICA 4	SN	R. Superior frontal gyrus	19, 12, 62	285
ICA 4	SN	R. Cerebellum Crus2	39, -53, -40	183
ICA 4	SN	L. Precuneus	-9, -62, 53	29
ICA 4	SN	L. Orbital frontal gyrus	-22, 45, -16	19
ICA 4	SN	L. Insula	-37, 13, 3	10
ICA 7	DMN	L. Superior frontal gyrus	-20, 34, 48	1706
ICA 7	DMN	L. Angular gyrus	-47, -63, 33	1480
ICA 7	DMN	L. Inferior temporal gyrus	-57, -21, -21	889
ICA 8	L. ECN	L. Dorsolateral prefrontal cortex	-44, 22, 27	2310
ICA 8	L. ECN	L. Inferior parietal gyrus	-35, -61, 46	1200
ICA 8	L. ECN	L. Inferior temporal gyrus	-54, -46, -15	74
ICA 10	R. ECN	R. Angular gyrus	46, -57, 41	2840
ICA 10	R. ECN	R. Dorsolateral prefrontal cortex	31, 24, 49	2294
ICA 10	R. ECN	R. Inferior temporal gyrus	63, -26, -18	627
ICA 10	R. ECN	R. Orbital frontal gyrus	35, 53, -3	378
ICA 10	R. ECN	L. Cerebellum Crus2	-39, -69, -45	151
ICA 13	DMN	Precuneus	0, -57, 34	6202

Abbreviations: SN: Salience Network; DMN: Default Mode Network; ECN: Executive Control Network; L: Left; R: Right; MNI: Montreal Neurological Institute; ROI: Region of Interest; ICA: Independent Component Analysis.

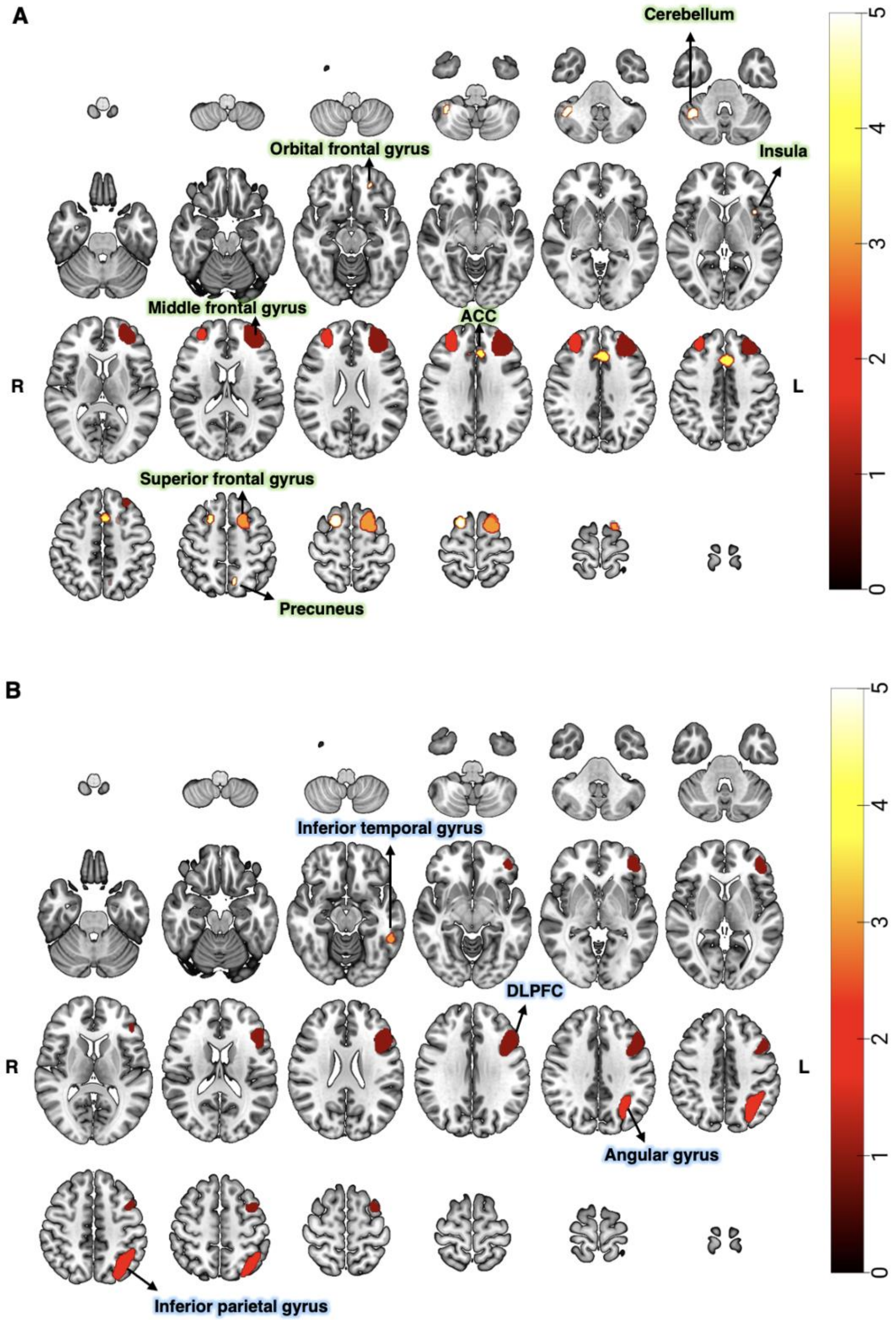
A
Cerebellum
Orbital frontal gyrus
Insula
Middle frontal gyrus
ACC
R
L
Superior frontal gyrus
Precuneus
0
1
2
3
4
5
B
Inferior temporal gyrus
DLPFC
R
L
Angular gyrus
Inferior parietal gyrus
0
1
2
3
4
5

C

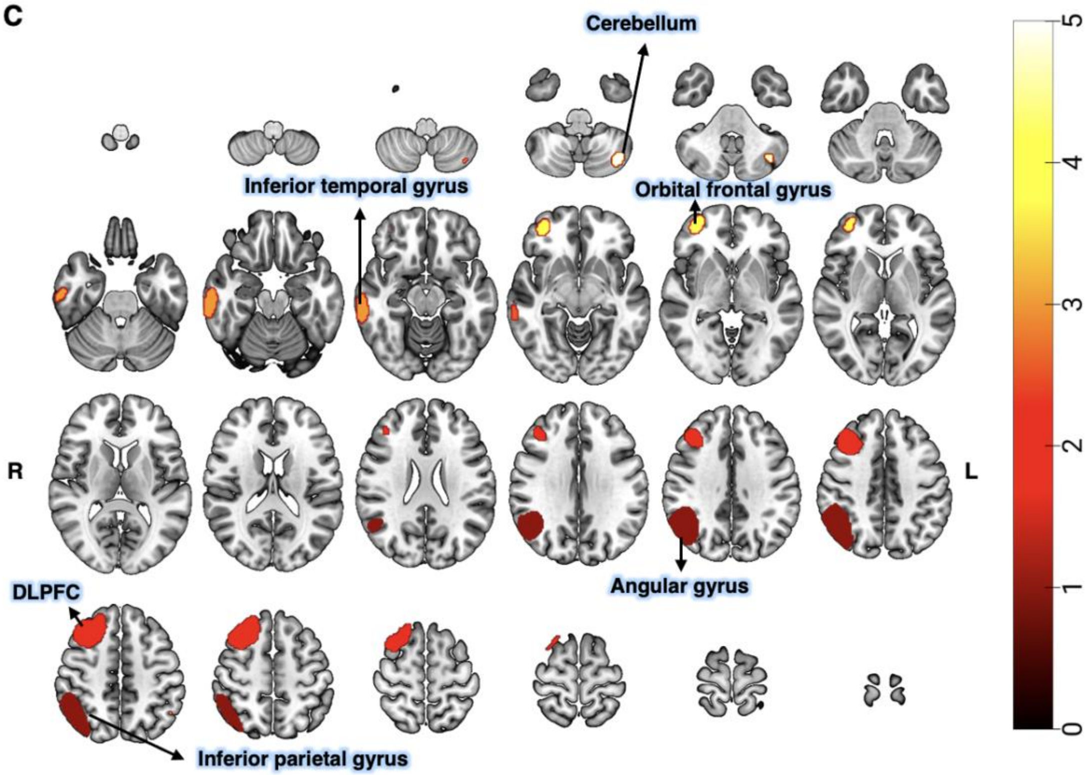
Cerebellum
Inferior temporal gyrus
Orbital frontal gyrus
R
L
DLPFC
Angular gyrus
Inferior parietal gyrus
5
4
3
2
1
0

D

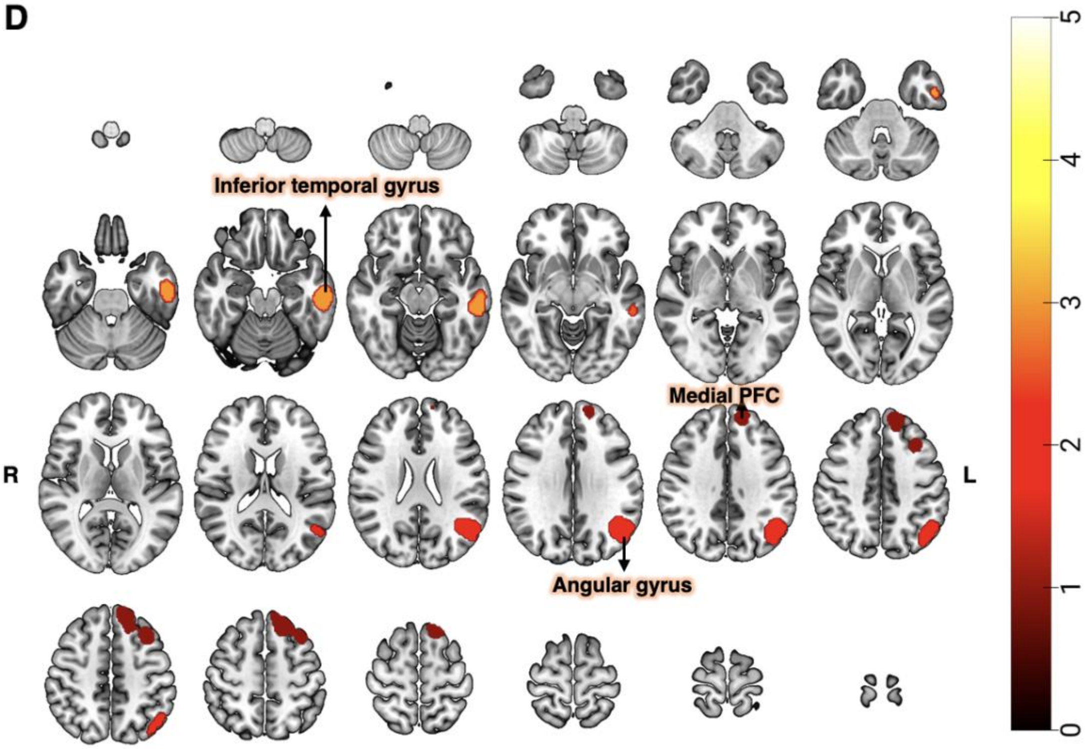
Inferior temporal gyrus
Medial PFC
R
L
Angular gyrus
5
4
3
2
1
0

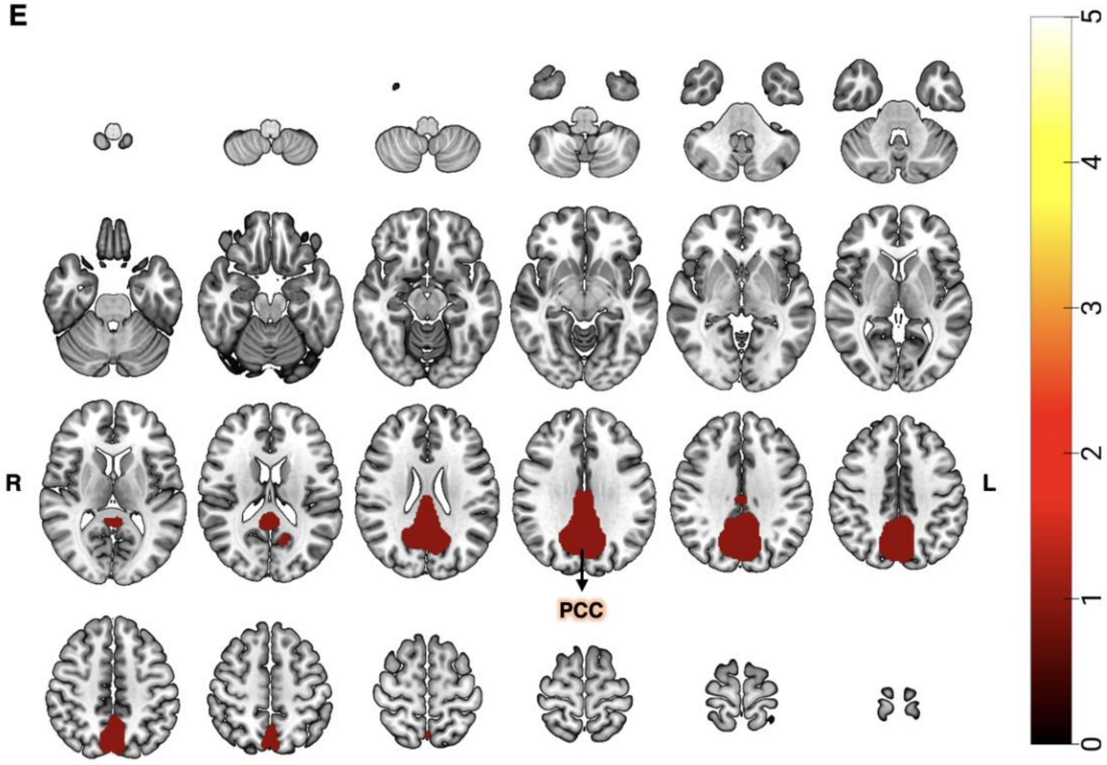

Supplementary Figure 1. ICA results. (A) Salience network, (B) Left ECN, (C) Right ECN, (D) DMN, (E) DMN.

Abbreviations: ACC: Anterior Cingulate Cortex; DLPFC: Dorsolateral Prefrontal Cortex; PCC: Posterior Cingulate Cortex; PFC: Prefrontal Cortex.

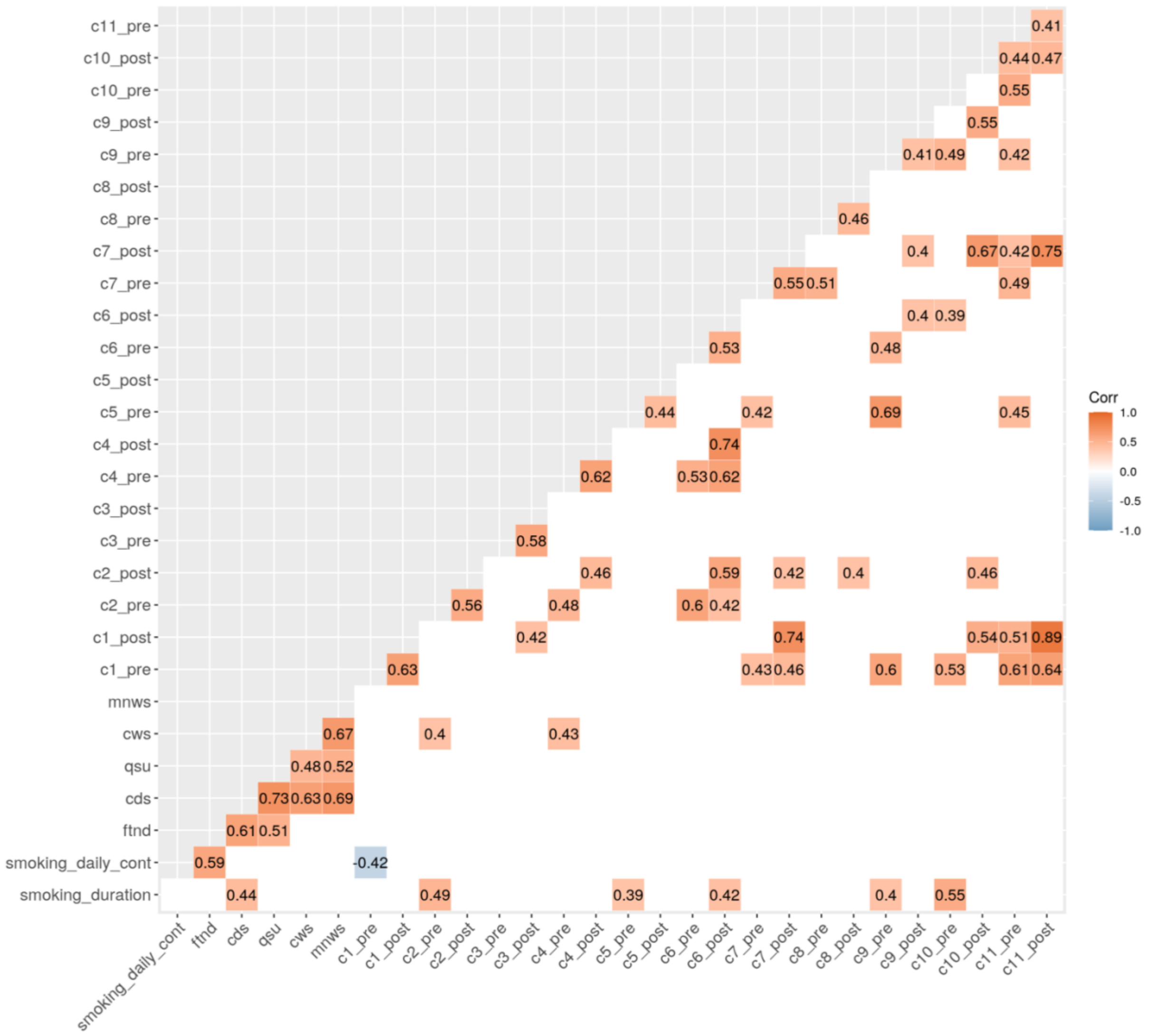

Supplementary Figure 2. Correlation plot of functional connectivity values from the significant clusters and smoking-related variables for the successful quit group.

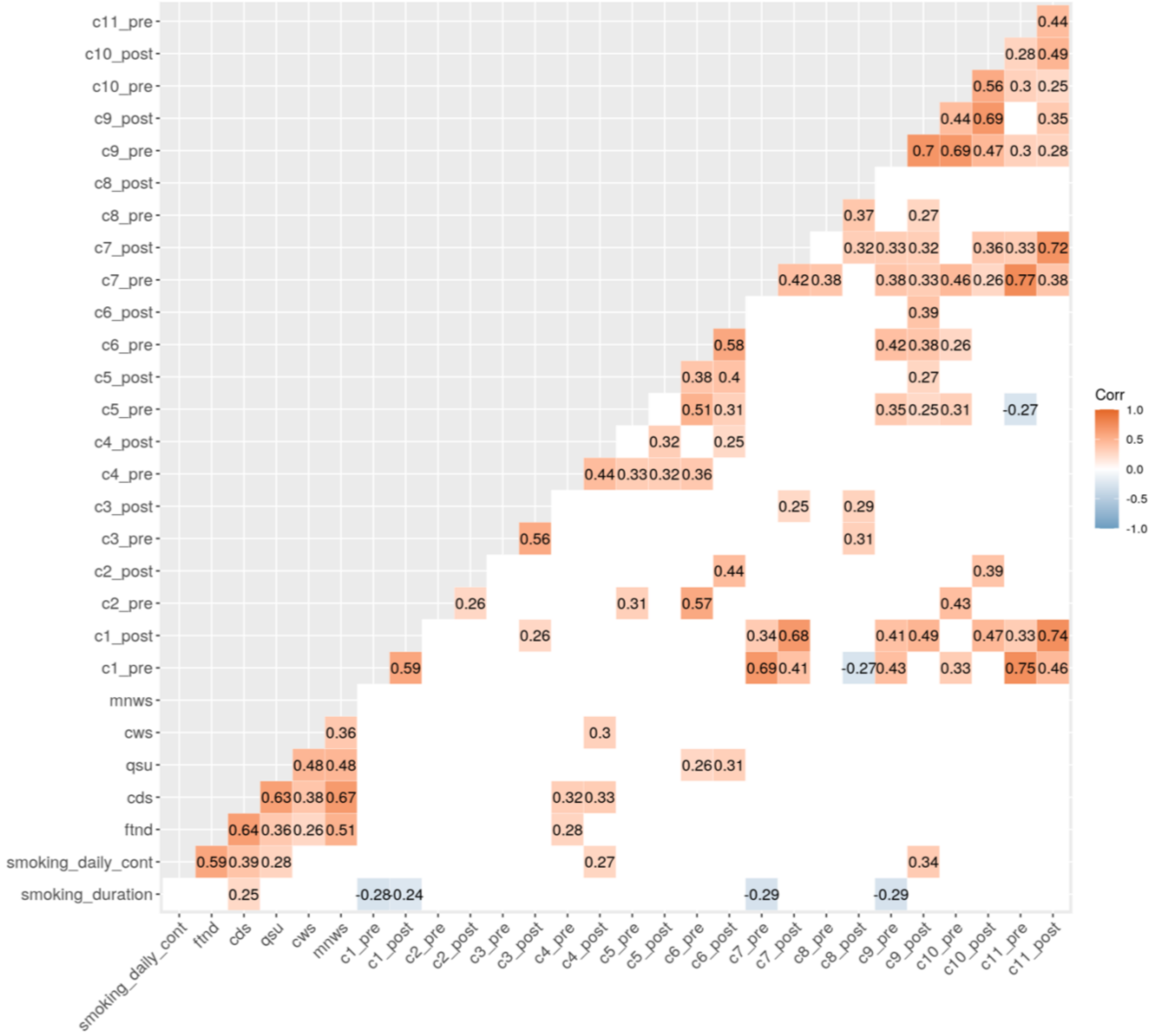

Supplementary Figure 3. Correlation plot of functional connectivity values from the significant clusters and smoking-related variables for the unsuccessful quit group.
